# Association between Serum Ferritin and Goitre in Iranian School Children

**DOI:** 10.3329/jhpn.v28i2.4883

**Published:** 2010-04

**Authors:** Mahin Hashemipour, Fahimeh Soheilipour, Ammar Hassanzadeh Keshteli, Mansour Siavash, Masoud Amini, Roya Kelishadi

**Affiliations:** ^1^ Isfahan Endocrine and Metabolism Research Center; ^2^ Medical Students Research Center, School of Medicine; ^3^ Vice Chancellery for Research; ^4^ Preventive Pediatric Cardiology Department, Isfahan Cardiovascular Research Center, Isfahan University of Medical Sciences, Isfahan, Iran

**Keywords:** Cross-sectional studies, Goitre, Iodine, Iron deficiency, Serum ferritin, Iran

## Abstract

Despite long-standing supplementation of iodine in Iran, the prevalence of goitre among general people remains high in some regions. The study investigated the role of iron status in the aetiology of goitre in school children in Isfahan, Iran. Two thousand three hundred and thirty-one school children were selected by multi-stage random sampling. Thyroid size was estimated by inspection and palpation. Urinary iodine concentration (UIC) and serum ferritin (SF) were measured. Overall, 32.9% of the children had goitre. The median UIC was 195.5 μg/L. The mean±SD of SF in the goitrous and non-goitrous children was 47.65±42.51 and 44.55±37.07 μg/L respectively (p=0.52). The prevalence of iron deficiency in goitrous and non-goitrous children was 9.6% and 3.1% respectively (p=0.007). Goitre is still prevalent in school children of Isfahan. However, their median UIC was well in the accepted range. Iron deficiency is associated with goitre in a small group of goitrous children. The role of goitrogens should also be investigated in this region.

## INTRODUCTION

Iodine-deficiency disorders (IDDs) are still a major health problem estimated to affect 750 million people worldwide (1). The spectrum of these disorders includes endemic goitre, hypothyroidism, endemic cretinism, and other congenital anomalies (2). One of micronutrients that can potentially influence IDDs is iron (3–5). Deficiencies of iron and iodine are major overlapping public-health problems in the developing world, and many children are at a high risk of both goitre and iron-deficiency anaemia (6). Iron deficiency adversely affects the physiology of thyroid, and supplementation of iron may improve the efficacy of oral iodized oil in goitrous children with iron-deficiency anaemia (7).

Endemic goitre is present in most parts of Iran (8), and iodine deficiency is considered a contributing factor for endemic goitre in the country (9). The National Committee for Control of IDD was formed in 1989 by the Ministry of Health and Medical Education. The production and distribution of iodized salt (40 mg of potassium iodide per kg of sodium chloride) began, and education of policy-makers, health personnel, and public on IDD was initiated in 1990. However, a survey of consumption of iodized salt showed that less than 50% of the population consumed iodized salt in 1993 with the mean urinary iodine of 5.0–8.2 μg/dL. Therefore, the first law requiring the mandatory iodization of all salts for household use was promulgated in 1994 (10). Isfahan is a city in central part of Iran with an approximate population of 2,000,000. The prevalence of goitre in Isfahan was estimated to be 92% in girls and 85% in boys in 1989 (11). Results of another study in 1997 showed that the prevalence of goitre among children aged 6–18 years in Isfahan was 62% (12).

The present study was carried out to estimate the prevalence of goitre and status of iodine and investigate the role of iron deficiency as a possible contributor to endemic goitre in school children of Isfahan, 15 years after the initiation of salt-iodization programme.

## MATERIALS AND METHODS

This cross-sectional study was performed on school children in Isfahan in 2005. Subjects were enrolled by multistage cluster random sampling (n=2,331). We excluded children with a history of exposure to radioactive iodine, thyroid surgery, or significant underlying disease, such as cardiopulmonary, liver or renal problems based on available medical records and interviewing parents and teachers.

Two endocrinologists performed goitre grading according to the classification of the World Health Organization/United Nations Children's Fund/International Council for the Control of Iodine Deficiency (WHO/UNICEF/ICCIDD) (1):

Grade 0: No palpable or visible goitre.

Grade 1: A goitre that is palpable but not visible when the neck is in normal position (i.e. the thyroid is not visibly enlarged).

Grade 2: A swelling in the neck that is clearly visible when the neck is in normal position and is consistent with an enlarged thyroid when the neck is palpated.

Written informed consent was taken from parents of all children. The Ethics Committee of the Isfahan Endocrine and Metabolism Research Center approved the study. A trained staff member drew a venous blood sample in a sitting position. The blood samples were transported on dry ice to the reference laboratory of the Isfahan Endocrine and Metabolic Research Center where these were stored at −70 °C until analysis. Urine samples were also collected for measuring iodine excretion. All blood and urine assays were performed within a median of 26 hours after sampling. The same person performed each assay using the same method.

Urine iodine concentration (UIC) was measured by the digestion method based on a modification of Sandell-Kolthoff reaction (1, 13). Serum ferritin (SF) was measured using immunoradiometric assay. Iron deficiency was defined as SF of <15 μg/L. Serum T4 was measured by radioimmunoassay (Iran Kavoshyar Co., Tehran, Iran). Concentration of TSH in serum was measured using immunoradiometric assay (Iran Kavoshyar Co., Tehran, Iran). The normal range of T4 was 4.5–12 μg/dL, and for TSH (thyroid-stimulating hormonic), it was 0.3–3.9 mU/L. Anti-thyroglobulin antibody (anti-Tg Ab) and anti-thyroperoxidase antibody (anti-TPO Ab) were measured by Rapid ELISA (Genesis Diagnostics, Littleport, UK). Intra- and inter-assay coefficient of variation for anti-Tg Ab was <12%, and for anti-TPO Ab, it was 7% and 5% respectively. Anti-Tg and anti-TPO concentrations of over 100 IU/mL and 75 IU/mL respectively were considered to be positive.

### Analysis of data

Quantitative variables are presented as mean±standard deviation (SD). Normality of data distribution was assessed with Kolmogorov-Smirnov test. Independent sample *t*-test and one-way analysis of variance were used for comparing measurements in different groups. Parameters not normally distributed were compared by Mann-Whitney test. The prevalence of iron deficiency between goitrous and normal children was compared by chi-square test. Pearson's correlation was used for finding correlation between SF and different quantitative variables; p value of <0.05 was considered significant. All analyses were performed using the SPSS software (version 15) (SPSS Corp, Chicago, IL, USA).

## RESULTS

Two thousand three hundred and thirty-one school children were enrolled in the study, with a female-to-male ratio of 1.60. Their age ranged from six to 13 years. The mean age±SD was 9.39±1.18 years for girls and 9.47±1.12 years for boys. Overall, 32.9% of the children (n=767) were classified as goitrous (Table 1). The prevalence of goitre among the girls was 32.4%, and 33.7% of the boys were goitrous (p=0.51).

**Table 1.** Thyroid size determined by inspection and palpation in school children of Isfahan, Iran

Subjects	Thyroid size			
	No.	Grade 0 (%)	Grade 1 (%)	Grade 2 (%)
Boys	898	66.3	27.3	6.4
Girls	1,433	67.6	29.0	3.4
All	2,331	67.1	28.3	4.6

Grade 0=No palpable or visible goitre;

Grade 1=A goitre that is palpable but not visible when the neck is in normal position;

Grade 2=A swelling in the neck that is clearly visible when the neck is in normal position and is consistent with an enlarged thyroid when the neck is palpated

UIC was measured in 454 randomly-selected children. The mean±SD and median UIC were 220.66±17.33 μg/L and 195.50 μg/L respectively. Seventy-two children (15.8%) had UIC<100 μg/L, and 3.7% had UIC <50 μg/L. One hundred and sixteen children (25.6%) had UIC between 200 and 300 μg/L, and 23.8% had UIC of more than 300 μg/L. UIC in the goitrous (n=152) and non-goitrous (n=302) children did not differ significantly. Comparing the mean UIC in the goitrous and non-goitrous children based on different sex groups did not show any significant difference either.

Ninety-four Grade 2 goitrous children (51 boys, 43 girls) and 326 non-goitrous children (149 boys, 177 girls) were randomly selected as cases and controls respectively for the measurement of SF.

The mean±SD of SF in the goitrous and non-goitrous children was 47.65±42.51 μg/L and 44.55±37.07 μg/L respectively (p=0.52). The SF level in the goitrous and non-goitrous girls was 42.53±36.23 μg/L and 46.76±45.20 μg/L respectively (p=0.57). SF in the goitrous and non-goitrous boys was 51.96±47.03 μg/L and 41.93±23.99 μg/L respectively (p=0.15). In the goitrous and non-goitrous groups, there were nine (9.6%) and 10 (3.1%) children with iron deficiency respectively [odds ratio (OR) 3.35, 95% confidence interval (CI) 1.32–8.50, p=0.007)]. Children in the first distributional quartile of SF concentration had a lower UIC than children in the fourth quartile (189.66±104.60 vs 227.54±122.68 μg/L, p=0.02). SF correlated with UIC (r=0.17, p=0.001). It did not, however, correlate with T4 and TSH levels.

Serum TSH and T4 were measured in 485 randomly-selected children. Six (1.2%) children had subclinical hyperthyroidism, and 82 (16.9%) had subclinical hypothyroidism. Clinical hyper- or hypothyroidism was not detected in any children tested. The goitrous children had significantly lower T4 levels than the non-goitrous ones (8.20±1.66 vs 8.84±1.48 μg/dL, p<0.001). There was no significant difference between TSH levels in the goitrous and non-goitrous children. The mean SF levels did not differ significantly between euthyroid and subclinically-hypothyroid children. Iron-deficient children had a higher prevalence of subclinical hypothyroidism than iron-sufficient ones (31.6% vs 17.1%, p=0.24).

While one iron-deficient child (10.5%) had positive anti-Tg Ab, 5.8% (n=23) of iron-sufficient children had positive anti-Tg Ab (p=0.40). The prevalence of positive anti-TPO Ab was higher in children with iron deficiency than iron-sufficient ones (10.5% vs 4.8%, p=0.26).

The mean serum TSH, T4, UIC, and thyroid autoantibodies in subjects with and without iron deficiency did not differ significantly (Table 2). There was also no significant difference in these variables by different SF concentration quartiles (Table 3).

**Table 2.** Serum levels of different variables in children with and without iron deficiency in Isfahan, Iran

Subjects	TSH (mU/L)	T4 (μg/dL)	Anti-TPO Ab (IU/mL)	Anti-Tg Ab (IU/mL)	UIC (μg/dL)
Iron-deficient (n=19)	3.04±1.41	8.25±1.46	92.53±252.06	87.49±286.32	19.95±14.47
Iron-sufficient (n=401)	2.85±2.76	8.72±1.55	27.57±109.73	56.40±319.80	21.84±11.56
Significance	NS	NS	NS	NS	NS

NS=Not significant;

Tg=Thyroglobulin;

TPO=Thyroperoxidase;

TSH=Thyroid-stimulating hormone;

UIC=Urinary iodine concentration

**Table 3.** Concentration of different variables based on quartile of SF in Grade 2 goitrous and non-goitrous school children of Isfahan, Iran

SF (μg/L)	TSH (mU/L)	T4 (μg/dL)	Anti-TPO Ab (IU/mL)	Anti-Tg Ab (IU/mL)	UIC (μg/L)
SF≤27.6	2.65±1.48	8.58±1.63	37.29±130.82	29.55±128.66	189.66±104.60
27.6<SF≤38.3	3.02±1.50	8.58±1.52	14.31±32.51	21.69±67.62	227.07±107.80
38.3<SF≤50.7	3.11±4.81	8.68±1.42	48.62±164.57	137.45±539.77	225.09±128.07
SF>50.7	2.62±1.40	8.94±1.59	21.54±109.12	42.05±292.15	227.54±122.68
Significance	NS	NS	NS	NS	NS

NS=Not significant;

SF=Serum ferritin;

Tg=Thyroglobulin;

TPO=Thyroperoxidase;

TSH=Thyroid-stimulating hormone;

UIC=Urinary iodine concentration

## DISCUSSION

According to the present study, the prevalence of goitre among school children in Isfahan has decreased from about 89% in 1989 (11) and 62% in 1997 (12) to 32.9% in 2005. This implies that iodine deficiency has been the most important cause of endemic goitre and also shows the effective role of the legislation for salt iodization in controlling goitre. However, goitre is still endemic in this area and is a severe public-health problem according to the WHO/UNICEF/ICCIDD-recommended criteria (1). According to the criteria, the indicator of elimination of iodine deficiency is a median value for UIC of 100 μg/L, and UIC should not be below 50 μg/L in more than 20% of samples (1). In the study population, the median UIC was 195.50 μg/L, and 3.7% of the population had UIC <50 μg/L, implying that there is no biochemical iodine deficiency in the overall population. More than 25% of the study children had iodine intake more than adequate, and 23.8% had excessive iodine intake. This indicates the risk of iodine-induced hyperthyroidism within 5–10 years after the introduction of iodized salt to susceptible groups (1). It was reported that, after prophylaxis with iodine salt in Zaire, 14% of patients had undetectable serum TSH values (14). In the present study, 1.2% of the children had subclinical hyperthyroidism, and there were no case of clinical hyperthyroidism. We suggest that the iodine content of salt in this region be monitored at regular intervals.

In areas with iodine-deficient people, multiple nutritional and environmental influences may contribute to the prevalence and severity of IDD. We showed that the school children with goitre in Isfahan had lower serum selenium levels than non-goitrous ones (15). However, there was no significant difference in concentration of serum retinol as an indicator of vitamin A status between goitrous and non-goitrous school children in Isfahan (16).

There are limited reports on interaction between the goitre rate and the iron status (17). In the Philippines, there was no difference in goitre rate between anaemic and non-anaemic subjects (18). In two studies in Iran and Ethiopia, no correlation was found between the iron status and the goitre rate or thyroid hormone levels (19, 20). In a clinical trial on goitrous children with and without iron deficiency, Zimmermann *et al*. found that the therapeutic response to oral iodine was impaired in goitrous children with iron-deficiency anaemia, suggesting that the presence of iron-deficiency anaemia in children limits the effectiveness of iodine-intervention programmes (3). In another trial, addition of encapsulated iron to iodized salt improved the efficacy of iodine in goitrous children with a high prevalence of anaemia (21). In the present study, we investigated the role of iron deficiency as a contributor to endemic goitre in school children in Isfahan. Although the mean SF level in the goitrous and non-goitrous children did not differ significantly, the goitrous children had a higher iron-deficiency rate than the non-goitrous ones. A similar finding was reported from a recent study in Iran where iron deficiency was associated with an increased rate of goitre (22). The mechanism by which the iron status influences thyroid and iodine metabolism is unclear (23). Iron deficiency decreases plasma T4 and T3 concentrations, reduces peripheral conversion of T4 to T3, and may increase concentrations of TSH (7, 24–26). Subjects with lower iron stores may have higher reverse T3 concentration (27). The two initial steps of thyroid hormone synthesis are catalyzed by thyroperoxidases that are dependent on iron (28). In addition, iron deficiency may alter the control of thyroid physiology in the central nervous system and modify nuclear T3 binding (24, 29). In the present study, there was no significant difference in the mean TSH and T4 levels between the iron-deficient and the iron-sufficient subjects. This is in agreement with a previous study by Azizi *et al*. (17) and in contrast to another study by Dabbaghmanesh *et al*. in which iron-deficient patients had a significantly higher TSH level and lower free T4 concentrations than those with a normal SF level (22). However, in our study, children in the first distributional quartile of SF had lower UIC levels than children in the fourth distributional quartile of SF, and this may be a possible explanation for the higher rate of goitre in the iron-deficient subjects. The impairment of thyroid peroxidase (TPO) activity may also influence thyroid metabolism. Iron-deficient rats had sharply reduced TPO activity (23). Although, in the present study, we did not determine the TPO activity, the iron-deficient children had higher anti-TPO Ab levels than iron-sufficient ones.

The main limitation of our study was that we categorized participants into goitrous and non-goitrous groups by inspection and palpation. It has been stated that, in areas of mild-to-moderate IDD, the sensitivity and specifity of palpation are poor (30). Classification of children into different goitre groups would have been more accurate, had we used thyroid ultrasonography instead of inspection and palpation. The evaluation of body iron status in our study was based only on the SF level. It would have been better to use other iron status parameters, such as serum iron, total iron-binding capacity, serum-soluble transferrin receptor, or transferrin concentration besides SF level. Lack of any statistically significant difference between SF levels in goitrous and non-goitrous school children could be attributed to the small sample size which is another limitation of the present study. SF can be falsely elevated during an infection. This can be adjusted with serum C-reactive protein (CRP) levels, a marker of infection. Although we did not measure CRP, the study children were free of any clinical infection when blood sampling was done.

We have shown that goitre is still a public-health problem in Isfahan. Iron deficiency is associated with goitre only in a small proportion of goitrous children, and these children may benefit from iron supplementation. Other factors, such as goitrogens or autoimmunity, may have a role in the still high prevalence of goitre in school children of Isfahan.

## ACKNOWLEDGEMENTS

The Bureau for Research, Isfahan University of Medical Sciences, funded the study. The authors are thankful to the authorities of the provincial and local education offices, all the staff working with the project, and students and their parents for their cooperation.
